# Understanding shame, guilt, embarrassment and pride: a systematic review of self-conscious emotions

**DOI:** 10.3389/fpsyg.2025.1678930

**Published:** 2025-11-12

**Authors:** Diksha Singh, Braj Bhushan

**Affiliations:** Indian Institute of Technology Kanpur, Kanpur, India

**Keywords:** self-conscious emotions, guilt, shame, embarrassment, pride

## Abstract

Self-conscious emotions play a crucial role in social functioning, moral behavior, and identity formation. Their inherent complexity, necessitating self-awareness and social evaluation, presents significant challenges for empirical research. This systematic review identifies methodological issues and gaps in experimental research related to the affective traits (valence and arousal), physiological and neural correlates, nonverbal cues, and cross-cultural variations of self-conscious emotions. A comprehensive search was conducted for peer-reviewed empirical studies published between 2000 and 2024 across databases such as PubMed, PsycINFO, Scopus, and Web of Science, adhering to PRISMA 2020 guidelines. Inclusion criteria focused on studies examining self-conscious emotions through physiological, neurological, behavioral, or cross-cultural approaches. A total of twenty-three studies were included, revealing variability in the valence and arousal profiles of self-conscious emotions, with distinct physiological and neural correlates identified in regions such as the medial prefrontal cortex, anterior cingulate cortex, and default mode network. Nonverbal cues, including posture, blushing, and gaze aversion, exhibited differences among emotions. Cross-cultural studies highlighted both universal and culturally specific patterns in the elicitation and expression of these emotions. However, limitations such as challenges in experimental induction, ecological validity, and construct operationalization hinder the generalizability and replicability of findings. The predominance of Western-centric studies further restricts cross-cultural applicability. Overall, self-conscious emotions remain underexplored compared to basic emotions, primarily due to methodological and ethical challenges. Future research should prioritize standardized definitions, ecologically valid designs, multimodal measurements, and diverse cultural samples to deepen the understanding of these complex emotions and their implications in clinical, social, and developmental contexts.

## 1 Introduction

As per Haidt (2003) classification of moral emotions, shame, guilt, embarrassment, and pride are a part of self-conscious emotions (SCEs) family, which are essential to social behavior and human moral functioning. In contrast to basic emotions that are triggered by outside stimuli (Tangney and Dearing, 2002; Tracy and Robins, 2004), SCEs originate from self-evaluative processes and necessitate a sense of self and the capacity to internalize social norms These feelings are essential to self-control and interpersonal relationships because they are usually elicited when people believe they have met or transgressed significant social or moral norms (Lewis, 2000).

Despite having a similar origin in self-awareness, SCEs are thought to have quite different affective characteristics. For example, guilt is more specific to one’s actions and usually stimulates reparative behavior (Baumeister et al., 1994), whereas shame is frequently linked to a general negative assessment of oneself and a desire to hide or withdraw (Tangney et al., 1996). Despite being frequently associated with these feelings, embarrassment is more ephemeral and is brought on by small social infractions (Keltner and Buswell, 1997). Compared to negative self-conscious emotions, pride has gotten less empirical attention. However, recent researches have distinguished between hubristic pride, which is linked to narcissism and arrogance, and authentic pride, which is linked to achievement and prosociality (Tracy and Robins, 2007; Weidman et al., 2016; Mercadante et al., 2021). It is essential to comprehend these two facets of pride in order to recognize both its adaptive and maladaptive functions in social interaction and self-evaluation.

### 1.1 Circumplex model of affect

The circumplex model (Russell, 1980) suggests that all affective states emerge from cognitive interpretations of fundamental neural sensations, driven by two distinct neurophysiological systems: valence and arousal. Two-dimensional models of affective experience have been developed through extensive analysis of the correlations between emotional experiences, utilizing statistical techniques such as multidimensional scaling and factor analysis on subjective reports of emotional words, facial expressions, and personal experiences (Larsen and Diener, 1992). Some conceptualisations of these aspects include approach and withdrawal (Lang et al., 1998), tension and energy (Thayer, 1990), positive and negative affect (Watson et al., 1999), and valence and arousal (Russell, 1980).

Circumplex theorists describe fear as a neurophysiological state characterized by increased arousal in the central nervous system coupled with negative valence. Cognitive interpretations of these physiological activity patterns that take place in response to evoking stimuli give rise to the subjective sensation of dread. To identify neurophysiological changes in the valence and arousal systems, cognitive interpretations play a crucial role in organizing these changes in relation to eliciting stimuli, past experiences, behavioral responses, and semantic knowledge (Russell, 2003). Consequently, emotions can be understood as the outcome of a dynamic interplay between cognition and neurophysiological shifts within the valence and arousal systems, primarily governed by subcortical structures.

Extensive research has repeatedly identified biological correlates for the two dimensions of emotional experience predicted by psychometric studies. Studies have shown a relationship between skin conductance and heart rate with subjective arousal ratings (Lang et al., 1993). Similarly, functional magnetic resonance imaging has revealed a correlation between signal intensity in the visual cortex and arousal ratings when viewing emotional images (Bradley et al., 2003). Subjective ratings of arousal correlate with increase in cerebral activation in EEG studies (Keil et al., 1999).

Valence ratings have likewise been linked to facial electromyographic measurements of the corrugator and zygomatic muscles (Cacioppo et al., 1986; Lang et al., 1993). Corrugator muscle activity gradually increases with negative valence ratings, independent of the specific affective state reported by participants, while zygomatic muscle activity rises progressively with positive valence ratings.

Interestingly all these classic psychometric studies involve basic emotions, especially fear, anger, and sadness. It is well known that shame, guilt, and embarrassment have negatively valence while pride has positive valence, but it lacks empirical evidence. While no evidence for arousal levels of these self-conscious emotions is known till date. Studies like Bhushan et al. (2020a) have tried to establish valence and arousal for guilt, shame and remorse and found it to be relative. For instance, they found in their storyboard-based study that same scenario can elicit guilt, shame or remorse depending on the valence perceived by the individual. Positive factor loads in principal component analysis (PCA) were associated with guilt, while negative factor loads corresponded to shame and remorse. This indicates that guilt is experienced with relatively more positive or reparative potential whereas shame and remorse are experienced more negatively closer to distress, alienation, and self-condemnation. Thermographic study by this team showed distinct thermal signatures for shame, guilt, and remorse (Bhushan et al., 2020b). While guilt involves greater somatic arousal compared to shame and remorse particularly in cheek and mouth regions, shame’s arousal was found to be more concentrated in the eye region. Although these studies give some insight about the valence and arousal of shame and guilt, embarrassment and pride are still far from clear. Hence, more experimental studies are required to establish valence and arousal dimensions for self-conscious emotions.

### 1.2 Non-verbal expressions of self-conscious emotions

Judgment studies have shown that self-conscious emotions such as pride, shame, and embarrassment are expressed through distinct nonverbal cues that are universally recognized across cultures. This suggests that these expressions may be universal rather than merely culturally constructed gestures (Haidt and Keltner, 1999; Tracy and Robins, 2004, 2007). Typically, recognition studies ask participants to identify the emotion portrayed by posing facial and body expressions. Although this methodology has been criticized, the expression is deemed valid if the degree of agreement surpasses what would be predicted by chance (Russell, 1994). The claim that pride and shame are universal is further supported by the observation that members of remote, preliterate cultures experience them (Tracy and Robins, 2008).

Both systematic observational studies and anecdotal reports that capture naturalistic emotional behavior have been used to derive these nonverbal expressions (Keltner, 1995; Lewis et al., 1992). Self-conscious emotions frequently necessitate the examination of extra non-facial cues, such as head movement, posture, and gestures, which are not entirely captured by the Facial Action Coding System (FACS; Ekman and Friesen, 1978), in contrast to basic emotions, whose facial expressions can be accurately described using FACS.

One major benefit of concentrating on nonverbal cues is that they can get around the drawbacks of self-report instruments, which rely on people being conscious of and open to sharing their emotional experiences. Particularly for socially sensitive emotions like shame, these requirements are frequently not fulfilled in practice (Tangney and Dearing, 2002; Shaver and Mikulincer, 2005). Furthermore, guilt and shame are often confused with one another (Tangney and Dearing, 2002). On the other hand, because they are less consciously controlled, nonverbal behaviors might offer a more accurate indication of emotional states (Ekman, 2003). However, they are transient, frequently necessitate video recording, and require a lot of work to analyse. Therefore, to improve the validity and depth of emotion research findings, it is advised to combine both self-report and observational methods.

### 1.3 Cross-cultural findings

The debate over whether emotion is universal or socially constructed has been a central topic in psychological research (Elfenbein et al., 2002; Mesquita et al., 1992; Russell, 1994). Some scholars argue that emotions are universally experienced and largely rooted in biological processes (Buck, 1998; Izard, 1994). Ekman (1971) further proposed that emotions are primarily genetically determined and expressed similarly across diverse cultures and nations. He suggested that individuals tend to feel positive emotions in favorable situations and negative emotions in response to adverse circumstances, a pattern observed across most basic emotions.

In cross-cultural psychology, culture is defined as the shared elements that shape perception, belief systems, evaluation, communication, and behavior among individuals who share a common language, historical period, and geographic location (Triandis, 1996). Western cultures tend to be associated with high-arousal emotions, while Eastern cultures are linked to low-arousal emotions (Tsai, 2007). These differences stem from the contrasting characteristics of individualist and collectivist cultural frameworks. Studies suggest that collectivist cultures place greater emphasis on shame and social harmony, while individualist cultures provide more value to guilt and personal responsibility (Mesquita and Walker, 2003; Eid and Diener, 2001). The experience and expression of pride also differ across cultures, with some viewing it as a sign of confidence and others as a social faux pas (Stipek, 1998). The majority of Western academics identify pride as the positive antithesis of shame and list shame, guilt, and embarrassment as the main self-conscious emotions (Lewis, 1971; Tangney and Fischer, 1995). However, most anthropologists note that non-Western distinct civilizations have different perspectives on things. In most Asian cultures, shame and embarrassment are not distinguished; instead, they are combined into a single culturally central emotion that includes social fear, shyness, and modesty in addition to what seems to be shame and embarrassment (Abu-Lughod, 2016; Fessler, 2004; Levy, 1983; Menon and Shweder, 1994; Russell, 1991). Furthermore, it has been proposed that shame and embarrassment are greatly developed in some non-Western cultures, but guilt is either non-existent or culturally unelaborated (Benedict, 1946; Levy, 1983). This cultural difference becomes understandable when one realises that self-conscious emotions are crucially dependent on two of the social life’s most culturally variable aspects one’s perception of oneself as independent or dependant (Markus and Kitayama, 2014) and whether society is hierarchical or egalitarian [Kitayama, (1991); also see Triandis et al. (1988)].

Given these theoretical and empirical complexities, there is a pressing need to systematically synthesize the literature on SCEs with a focus on their affective characteristics (arousal and valence) and the role of cultural variation. This review aims to fill that gap by analysing empirical studies on shame, guilt, embarrassment, and pride reported in the past two decades, identifying patterns and gaps in the literature, and providing a foundation for future research and clinical applications.

## 2 Methods

### 2.1 Literature search

A comprehensive search was conducted across the following databases— PubMed, PsycINFO, Scopus, Web of Science, and Google Scholar. The search strategy employed a combination of keywords and Boolean operators: “self-conscious emotions” OR “guilt” OR “shame” OR “embarrassment” OR “pride” AND “valence” OR “arousal” “affect” OR “physiology.” Search filters were applied to restrict results to human studies, peer-reviewed journals, and publications in English from 2000 to 2024. All the studies were searched between March and April 2025.

### 2.2 Eligibility criteria and selection process

This review strictly included studies that (a) examined one or more self-conscious emotions— shame, guilt, embarrassment, and pride; (b) involved human participants; (c) reported empirical findings using either quantitative or mixed methods designs; and (d) were published in peer-reviewed journals between 2000–2024. Studies were excluded if they were non-empirical (eg., theoretical papers, opinion pieces), dissertations, non-English publications or reviewed articles, abstracts of conference proceedings, and editorials and if they involved psychiatric patients and delinquents or jail inmates as participants.

### 2.3 Data collection process

Articles were selected on the basis of their title and abstracts. After identification they were screened according to inclusion and exclusion criteria. The full texts of 23 shortlisted articles were manually reviewed to ensure compliance with eligibility criteria.

### 2.4 Data extraction

Data from empirical studies were extracted for authors (year), type of self-conscious emotion(s) studied, sample characteristics, measures/tools used, and findings. It is summarized in Table 1.

**Table 1:** Study characteristics and findings.

Authors (Year)	Self-conscious emotion	Sample characteristics	Tools/Measures/Stimuli	Findings
Hejmadi et al. (2000)	Shame/Lajya	95 participants (48 American college students from university of Wisconsin) (47 Hindu Indians from Bhubaneswar, Orissa).	Stimuli: Videotaped portrayals of emotions by trained classical Indian dancer (Natyasastra) Fixed Response to choose from a list of 10 emotions. Free-Response for naming of perceived emotion(s) For validation two Indian dance experts verified the authenticity of emotional portrayals	In the free-response format Indians were more accurate than Americans for the three indigenous emotions, *lajya, peace* and *heroism*. These results support the cultural specificity of certain self-conscious emotions and demonstrate that some emotions may not translate cleanly across cultures.
Harris (2003)	Shame and Guilt	720 individuals apprehended for driving with a blood alcohol of over 0.8 with mean age of 30 years and 76% were male.	Self-report measures of moral emotions, empathy, and anger/hostility	The study failed to find distinction between shame and guilt. Principal components analysis revealed three factors: shame guilt, embarrassment exposure, and unresolved shame Shame guilt was found to be associated with higher levels of empathy and lower levels of anger and hostility.
Takahashi et al. (2004)	Guilt Embarrassment	19 healthy right-handed Japanese subjects (10 men, mean age = 30.8 and 9 women mean age = 25.1)	Short stories with emotional content with self-report measure and fMRI	Guilt condition relative to neutral condition produced greater activations in the MPFC, left posterior STS, and visual cortex. Embarrassment condition produced greater activations in the MPFC, left posterior STS, left temporal cortex, left orbitofrontal cortex, right temporal cortex, left hippocampus and visual cortex. The evaluative process of embarrassment may be more complex than that of guilt.
Finger et al. (2006)	Guilt Shame Embarrassment	16 healthy individuals (gender/age not specified)	Stimuli: Emotion induction statements (script driven, instructed to imagine oneself in situation) Tool: fMRI	Moral transgression evoked strongest feelings of guilt followed by shame and embarrassment while when audience was present it increased shame and embarrassment ratings slightly but not guilt. Social transgression evoked strongest feelings of embarrassment followed by shame and guilt and audience significantly increased embarrassment, shame and to a lesser extent guilt. Ventrolateral and Dorsomedial prefrontal cortices were more active during moral transgression regardless of audience and social transgression only when an audience was present. Left amygdala showed increased activation in all scenarios where an audience was present regardless of transgression type.
Tracy and Robins (2008)	Pride	Study 1: 28 individuals (50% women; mean age 27 years) from Italy. Study 2: 39 individuals (68% women; mean age 46 years) from rural tribal villages near Toussianna. All were non literate Study 3: 38 undergraduate students (65% women) from university of California. Study 4: 211 undergraduates (60% women), randomly assigned to view 1 of 6 target types (by ethnicity and gender).	Study 1: photographs of 4 targets (2 Asian-American and 2 Caucasian-American; male and female) displaying posed expression of Pride, happiness, surprise, and contempt Study 2: photos of 4 targets (2 Caucasian American, 2 west African) displaying 8 emotional expressions validated by FACS coding. Study 3: Drawn figure of a character expressing each of 8 emotions. Ethnicity and gender were systematically manipulated across figures (African, Asian, Caucasian; male and female) Study 4: same drawn figure stimuli from study 3.	Study 1 Italian participants accurately recognized pride from static posed photos. Pride was recognized at similar or higher accuracy compared to basic emotions like happiness and surprise. Suggesting that pride has a cross-culturally recognizable nonverbal expression even in cultures wehre it may not be highly emphasized. Study 2: Pride was recognized above chance by individuals from a small-scale, non-Western and non-Literate culture. Study 3: Participants accurately recognized pride across all ethnicities and genders of the drawn figure stimuli. No significant differences in recognition based on target’s ethnicity or gender. Study 4: Even when exposed to only one character across all emotion expressions, participants still recognized pride above chance. Suggests that pride expression is clearly distinct and identifiable, not reliant on exposure to multiple comparative expressions.
Darby and Harris (2010)	Embarrassment	54 undergraduates; Asian (23), Caucasian/white (6), Hispanic/ Latino (7), pacific islander (3), others (5)	Tobii 1750 Eye Tracker Bried fear of negative evaluation scale Interaction anxiety scale	Embarrassment appears to have different effects of facial information processing than social anxiety. Embarrassed participants fixated proportionally more on the eyes than control and also fixated proportionally less on other less emotionally informative areas of the face compared to controls
Drummond and Su (2012)	Embarrassment	67 women and 19 men; aged between 19–59 years.	Blushing Propensity Scale. Fear of Negative evaluation scale Social interaction anxiety Social phobia scales Laser doppler flowmetry with Mbf3d Laser Blood flow Monitor and biopac	Women showed greater increases in facial blood flow than men during task. Higher scores on the Blushing Propensity Scale and Social Interaction Anxiety Scale were associated with greater increases in facial blood flow. The study suggests that social anxiety influences beliefs about blushing and affects changes in facial blood flow.
Müller-Pinzler et al. (2012)	Embarrassment	54 female subjects, all German native speakers.	ECG, GSR, SKT PPG	Vicarious embarrassment elicited a pattern of increased autonomic activation, characterized by decreased heart periods and increased electrodermal activity. The study did not find evidence of blushing responses.
Somerville et al. (2013)	Embarrassment	69 healthy participants aged between 8.0 and 22.9 years recruited from New York City Metropolitan area	Self-report Emotion Ratings, Galvanic skin response and fMRI	Adolescents exhibited significantly heightened ratings of embarrassment in response to social evaluation compared to younger children and young adults. The peak embarrassment ratings were reported to occur around 17.2 years of age, indicating a developmental trend in self-conscious emotions. Skin conductance responses (GSR) showed that adolescents had lower rates of habituation (indicating sustained autonomic arousal) during social evaluation compared to adults. This suggests heightened sensitivity to social evaluations during adolescence, with significant differences observed especially in the first half of the experimental session. Functional MRI results indicated that the medial prefrontal cortex (MPFC) displayed notable activity during social evaluations, with distinct age-related patterns. The study found that MPFC activation exhibited non-linear changes across age groups, aligning with the behavioral findings of embarrassment and emotional arousal. The findings emphasized that social sensitivity, reflected both behaviorally (in terms of self-reported emotions) and physiologically (through GSR), is heightened during adolescence, potentially influencing motivation and emotional well-being in social contexts.
Zahn et al. (2014)	Guilt Pride	63 individuals (30 M, 30F; mean age 28.1 years)	Stimuli: Value-related moral sentiment task (VMST) (Zahn et al., 2014) Written description of positive or negative moral behaviors. Tool: Voxel-based morphometry (VBM) with DARTEL for analysis of grey matter volume. MRI	Pride-proneness was associated with reduced grey matter volume in left cuneus and right precuneus. In secondary analysis, greatwer fMRI responses to guilt in the subgenual cingulate cortex were associated with reduced GM volume in the right anterior dorsolateral prefrontal cortex and left superior temporal sulcus.
Gilead et al. (2016)	Pride Guilt	20 right-handed participants (14 women, mean age 25.9 years) from Tel-Aviv University. All were native Hebrew speakers.	fMRI Rating scales Stimuli: Scenarios	Negative self-conscious emotions were found to be associated with a dorsal activation and positive self-conscious emotion (pride) was associated with activation of ventral medial prefrontal cortex. Processing of self-conscious emotions regardless of their valence, activated the dACC and lateral-dorsal prefrontal cortex suggesting effortful cognitive control Negative emotions (guilt) showed stronger and broader brain activation than positive emotions (pride).
Weidman et al. (2016)	Pride	Study 1: 108 adults from greater Vancouver. Study 2: 1024 undergraduate students.	14-item Authentic and Hubristic Pride Scale (Tracy and Robins, 2007) 8-item New general elf-efficacy scale (Chen et al. 2001).	Study1: authentic pride promotes achievement-related behavior in the athletic domain. Study 2: all of the functional effects of authentic pride on achievement were stronger than those for hubristic pride, suggesting that the link between pride and achievement is primarily driven by authentic pride.
Sznycer et al. (2018)	Pride	Study 1: Data were collected from 1,458 participants across 16 countries using various recruitment methods (e.g., AMT, survey companies, social networks); ~6% were excluded due to failed attention checks. Study 2: Recruited via AMT, with final samples of 202. U.S. participants (mean age 38, 120 females) and 147 Indian participants (mean age 33, 50 females) after attention check exclusions.	Rating Scales, Scenarios	Pride consistently reflects how individuals are valued by others across cultures. This link is unique to pride and does not apply to other positive emotions. Pride appears to function as an evolved mechanism that encourages socially valued behavior and enhances social status, supporting the advertisement recalibration theory which views pride as both a motivator and signal of valued traits.
Cesare Cavalera et al. (2018)	Shame Guilt	Experiment 1: 76 female undergraduates (mean age 22 years) Experiment 2: 65 female participants	Stimuli: Autobiographical writing task for inducing guilt and shame. Working memory task: digit recall, manual tracking task. State shame and guilt scale (SGSS-8)	Both shame and guilt significantly impaired working memory performance. In both experiments performance dropped significantly after emotional writing compared to neutral group. Shame had a stronger negative effect than guilt on WM performance.
Gambin and Sharp (2018)	Guilt	117 inpatient adolescents (75 females and 42 males) Recruited from a private psychiatric hospital in a major metropolitan city in the southwestern US.	Basic empathy scale (BES) Beck Depression Inventory-II Test of self-conscious affect- Adolescent (TOSCA-A) Personal Feelings Questionnaire (PFQ-2)	Generalized guilt, contextual and generalized shame mediated the relation between affective empathy and depressive symptoms. Cognitive empathy was shown to be related most strongly to contextual guilt and was unrelated to depressive symptoms.
Bhushan et al. (2020a)	Guilt Shame Remorse	Phase I: 10 participants (8 males, 2 females; age 20–25) wrote personal experience of guilt, shame and remorse. Phase II: 14 participants (7 males, 7 females) rated narratives and illustrations for item equivalence. Phase III: 138 male undergraduates, mean age 20.9 years.	34 written scenarios later refined to 13 storyboard illustrations, each comprising 3 images 5-point Likert intensity scale	Principal Component Analysis and Multidimensional Scaling showed that the same scenario could evoke different emotions depending on the individual. Guilt scenarios clustered distinctly shame and remorse which overlapped more. The mapped perceived similarity and dissimilarity among these emotions and provided culturally grounded operational definitions.
Bhushan et al. (2020b)	Guilt Shame remorse	31 male participants; aged between 19–24 years (mean age 20.84)	Stimulus: storyboards of guilt, shame and remorse scenarios (Bhushan et al., 2020b) Tool: Thermal Imaging Camera	Guilt induced significantly higher facial temperature at cheeks and forehead than shame or remorse. Distinct thermal signatures were observed for guilt versus remorse and shame for certain scenarios.
Sznycer and Cohen (2021)	Pride	243 participants (143 females; mean age 39 years) in USA and 167 participants (55 females; mean age 29 years) in India Recruited via Amazon Mechanical Turk	25 brief hypothetical scenarios (socially valued acts or traits) developed by Sznycer et al. (2018). 6 experimental conditions and 1 control (valuation, pride feeling, communicate event, demand better treatment, invest in valued trait, pursue new challenges, and destroy evidence)	Pride feelings and motivations increase proportionally with how much a behavior is valued by others. Pride feelings, communication, investment, and goal pursuit correlated positively with one another. Patterns of pride responses and valuations were similar across cultures though stronger and more consistent in the USA
Kusano and Kemmelmeier (2022)	pride	Study 1: 329 undergraduate students at a large western US public university Study 2: 177 undergraduates Study 3: 328 participants (77% females) Study 4: 107 undergraduates	The Relived Emotion Task False-feedback interpersonal perception task Leadership aptitude test Self-rated Pride Feelings Big five Inventory (Rammstedt and John, 2007) 13-item Narcisstic Personality Inventory (Gentile et al., 2013)	Hubristic pride was harder to elicit with generally low-self reported ratings. Authentic pride correlated with positive affect, self-esteem, agreeableness, and conscientiousness. Hubristic pride correlated more strongly with negative affect, especially hostility, shame and aggression, raising doubts about its status as a positively valences emotion. The overall findings suggest that hubristic pride may not be discrete emotion and that a single-facet model of pride may be more appropriate
McGarity-Shipley et al. (2022)	Shame	15 young healthy non-smoking adults recruited from Queen’s University and the Kingston Community.	7-day physical activity recall ECG PANAS Experiential shame scale (ESS)	Findings tentatively suggest that, independent of hypothalamic–pituitary–adrenal axis activation and activation of inflammation, increased feelings of shame result in temporarily reduced endothelial function and might therefore have a transient negative impact on vasoprotection
Giorgetta et al. (2023)	Guilt Embarrassment Shame	163 participants 98 Italians (56 females; mean age 34.36 years) 65 Americans (32 females; mean age 34.66 years)	16 scenarios: 4 guilt, 4 shame and 8 embarrassment 10-point Likert rating scale Control scales: happiness, responsibility and realism Software: Jamovi for analysis	Guilt scenarios effectively elicited guilt and embarrassment scenarios effectively elicted embarrassment in both cultures. Italians distinguished more clearly between shame (vergogna) and guilt (colpa). Americans showed greater overlap between shame and guilt implying a cultural conflation of moral emotions Italians showed stronger associations between shame and embarrassment while Americans showed mixed correlations including shame-guilt links. The study highlights that emotion terms like “shame” and “vergogna” or “embarrassment” and “imbarazzo” are not direct equivalents across languages and cultures.
Muniak and Kulesza (2024)	Guilt	Study 1: 62 participants (48 women and 14 men) mean age of 26.05 Study 2: 93 local university student (50 women and 43 men) mean age of 36.15 Study 3: 100 participants (52 women and 48 men) mean age 35.11 All participants were local students from 2 universities in Poznan and Warsaw.	Guilt scale developed by Wojciszke and Baryła (2005)	Findings suggested that being mimicked might indeed increase feelings of guilt in certain contexts. The results indicated a trend where individuals felt a heightened sense of guilt when in the presence of mimicking behavior
Basu et al. (2024)	Shame Guilt Remorse	147 male undergraduates, aged 19–25 years; mean age 21.04 Native Indians	13 pictorial storyboards depicting real life scenarios for guilt, shame and remorse developed by Bhushan et al. (2020a) 5-point Likert Rating Scale	Openness to experience was positively associated with shame and negatively with guilt Neuroticism and extraversion predicted guilt. No personality trait significantly predicted remorse.

## 3 Results

### 3.1 Study selection

**Figure 1:**
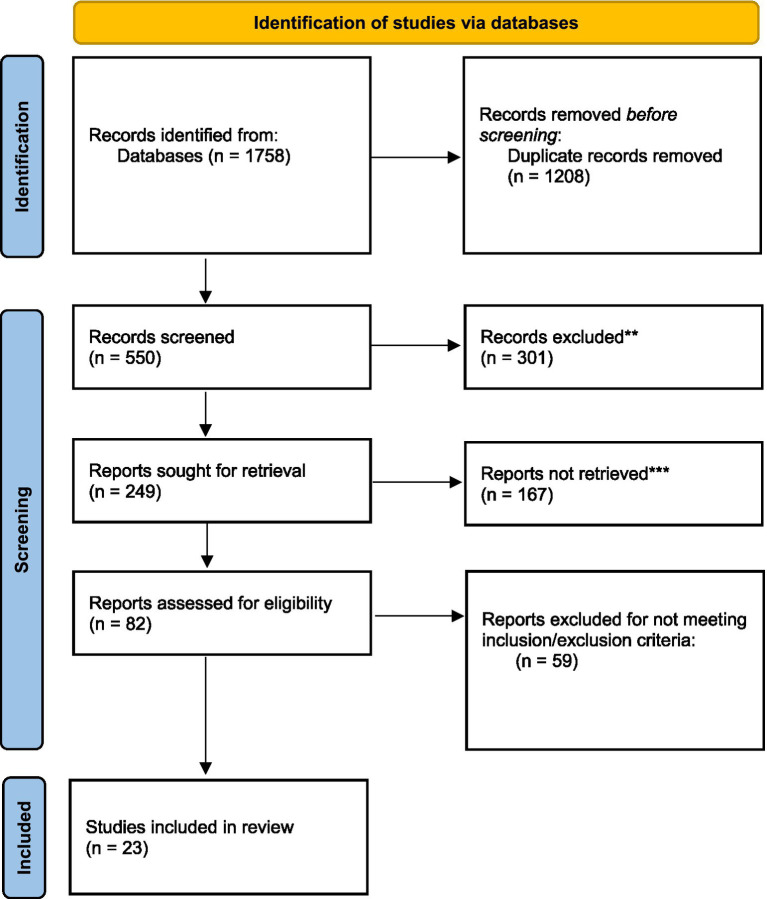
Flow diagram of identification process. **Full text was not available/ text was not in English. ***Reviews/conference papers/ dissertations.

A comprehensive review yielded a total of 1,758 studies, of which 550 were screened after duplicates were removed. After screening title and abstracts, 249 full-text articles were assessed for eligibility. Finally, 23 studies met inclusion/exclusion criteria and were included in this review. Figure 1 depicts the PRISMA 2020 flow diagram for study selection process.

### 3.2 Study characteristics

The studies included in this systematic review panned from 2000–2024 and utilized varied methodologies including eye-tracking, facial electromyography, fMRI, thermal imaging camera, behavioral tasks, and self-report measures. Participants ranged from young children to adults, with minimum sample size being 15 participants and maximum being 329 participants. Most of the studies (Darby and Harris, 2010; Keltner, 1995; Weidman et al., 2016; Cavalera et al., 2018; Kusano and Kemmelmeier, 2022; Basu et al., 2024) involved undergraduates as participants.

Broadly, the findings of these studies throw light on the basic nature of SCEs. They are succinctly summarized below.

#### 3.2.1 Shame

Shame is a self-conscious feeling that is closely related to both internal self-judgment and social evaluation. It exhibits notable cultural variability. Giorgetta et al. (2023) discovered that Italians distinguish shame from guilt more clearly than Americans, suggesting that cultural schemas have a significant impact on emotion differentiation. Shame has a distinct facial thermal profile compared to guilt and remorse (Bhushan et al., 2020b), and it has been demonstrated physiologically to impair endothelial function, affecting cardiovascular reactivity (McGarity-Shipley et al., 2022). More than guilt, shame impairs executive functioning, particularly working memory (Cavalera et al., 2018). Although shame is typically associated with neuroticism and low self-esteem, recent research shows a surprising positive correlation between it and openness (Basu et al., 2024). According to Somerville et al. (2013), adolescents increased neural sensitivity to social evaluation, especially in the medial prefrontal regions, causes them to display heightened shame. In contrast to guilt, shame is more difficult to identify facially (Keltner, 1995). In American populations, shame and guilt are frequently confused, but in Italians, they are more easily distinguished (Giorgetta et al., 2023).

#### 3.2.2 Guilt

Moral or interpersonal transgressions give rise to guilt, which is closely linked to reparative intent and sympathetic concern. As evidenced by its involvement in self-evaluation and moral reasoning, it activates the dorsal medial prefrontal cortex (dMPFC), anterior cingulate cortex (ACC), and posterior superior temporal sulcus (STS) (Takahashi et al., 2004; Gilead et al., 2016). Compared to shame, guilt has a less severe effect on working memory (Cavalera et al., 2018). By encouraging repentance, confession, and reparations, it fulfils an adaptive social function (Muniak and Kulesza, 2024; Giorgetta et al., 2023). It has been demonstrated that guilt clusters affectively apart from regret and shame (Bhushan et al., 2020a). In contrast to pride, guilt activates the dorsal MPFC instead of the ventral one (Gilead et al., 2016). Similar to shame, different cultures have different ways of recognising it and understanding it (Giorgetta et al., 2023).

#### 3.2.3 Embarrassment

Usually, social faux pas or norm violations that are noticed by others cause embarrassment. It is closely related to social evaluation contexts and the presence of observers (Finger et al., 2006). It causes physiological changes that are visible to the naked eye, like flushing and an increase in blood flow to the face (Drummond and Su, 2012). The anterior insula and medial prefrontal cortex (MPFC) are activated by both personal and vicarious embarrassment, according to neuroimaging studies, underscoring the emotional and self-reflective aspects of embarrassment (Müller-Pinzler et al., 2012). Typical nonverbal indicators of embarrassment include awkward smiling, face touching, and gaze aversion (Darby and Harris, 2010). Although embarrassment has unique characteristics, it is less well-known than basic emotions but can still be distinguished from shame and guilt (Keltner, 1995). According to Somerville et al. (2013), adolescents increased social sensitivity and MPFC activity cause them to report higher levels of embarrassment. In contrast to shame and guilt, embarrassment is more about social awkwardness than moral failure, and is physiologically more intense when someone else is around (Finger et al., 2006).

#### 3.2.4 Pride

Both literate and non-literate population acknowledge pride, a self-conscious emotion associated with accomplishment and social status, indicating that it has evolved to be conserved (Tracy and Robins, 2008). It has two subtypes: hubristic pride, which is connected to conceit and narcissism, and authentic pride, which is connected to competence and prosocial behavior (Weidman et al., 2016; Kusano and Kemmelmeier, 2022). Pride is in line with the brain’s valuation systems and reflects perceived social value (Sznycer et al., 2018; Sznycer and Cohen, 2021). Pride activates the left temporal pole and ventral MPFC, according to neuroimaging, suggesting positive self-referential processing (Gilead et al., 2016). According to Kusano and Kemmelmeier (2022), pride has a positive correlation with conscientiousness and self-esteem. Unlike guilt, pride causes the ventral MPFC to fire instead of the dorsal one (Gilead et al., 2016). The hubristic subtype is contentious because it shares characteristics with narcissistic and shame traits, making it difficult to classify as a completely positive emotion (Kusano and Kemmelmeier, 2022).

### 3.3 Affective characteristics

With the exception of pride, which typically has positive valence, all of the self-conscious emotions that were examined were consistently characterized by negative valence. Both shame and guilt have negative valence, but shame is more frequently linked to avoidance and withdrawal, whereas guilt is more frequently linked to approach-related tendencies like reparative behavior and interpersonal engagement (Tangney and Dearing, 2002; Tracy and Robins, 2007). Embarrassment frequently serves affiliative social functions and is generally negative, albeit less intense than shame or guilt (Keltner and Buswell, 1997; Miller, 1996). Pride and embarrassment are consistently associated with high levels of arousal, which are frequently accompanied by an increase in sympathetic nervous system activity (Tracy and Robins, 2008; Leary et al., 1996). While shame exhibit variable arousal responses— sometimes high, especially in socially evaluative contexts (Dickerson et al., 2004), but it also shows lower arousal in contexts where withdrawal or defeat is the primary response (Gruenewald et al., 2004; Keltner and Buswell, 1997).

### 3.4 Physiological correlates

Shame, guilt, and embarrassment all show physiologically elevated autonomic responses, such as increased skin conductance and heart rate (Leary et al., 1996; Kreibig, 2010). For every emotion, different neural correlates have been found. Shame has often been linked to activation in the anterior cingulate cortex (ACC) and medial prefrontal cortex (mPFC), reflecting social evaluative and self-referential processes, as well as the experience of social pain (Takahashi et al., 2004; Zahn et al., 2014). The temporoparietal junction (TPJ) and ventromedial prefrontal cortex (vmPFC), which are involved in moral cognition and empathy, are activated when someone feels guilty (Bzdok et al., 2012; Wagner et al., 2011). In socially awkward situations, embarrassment is more consistently associated with the insula and amygdala, indicating heightened emotional salience and self-conscious affect, although it shares activation with shame and guilt (Müller-Pinzler et al., 2012; Krach et al., 2016). Pride’s association with status enhancement and positive reinforcement is supported by the activation of reward-related regions like the nucleus accumbens and ventral striatum (Takahashi et al., 2004; Zahn et al., 2014). The idea of functionally differentiated emotion profiles is supported by the fact that each emotion involves additional unique networks, even though self-referential regions (like the mPFC) exhibit shared activity (Bastin et al., 2016).

### 3.5 Nonverbal indicators

Consistently, self-conscious emotions display unique, identifiable nonverbal behaviors. Slumped posture, head lowering, and gaze aversion are signs of shame that indicate disengagement and submission (Keltner and Buswell, 1997; Tracy et al., 2007). Although guilt does not have a distinct expressive signature, it is frequently expressed through reparative gestures and facial concern (Tangney et al., 1996). Appeasement signals that ease social tension, such as blushing, face touching, nervous laughter, and downward head movement, are consistently linked to embarrassment (Keltner, 1995; Miller, 1996). An enlarged chest, a high head, akimbo or raised arms, and a small smile are characteristics of pride that convey self-assurance and social standing (Tracy and Robins, 2004, 2007). These cues are discernible to observers from all cultural backgrounds and impact social assessments of warmth, competence, and morality (Tracy and Robins, 2008).

### 3.6 Cultural differences

The experience, manifestation, and interpretation of self-conscious emotions are greatly influenced by cultural factors. In contrast to individualistic cultures where shame is frequently perceived as maladaptive, shame is more frequently and constructively expressed in collectivistic cultures (such as East Asia), where it fosters social harmony and relational interdependence (Fessler, 2004; Wong and Tsai, 2007). Guilt is frequently more common in Western contexts and is more in line with individualistic values of personal responsibility and internal moral standards (Mesquita and Walker, 2003). Although everyone experiences embarrassment, the circumstances and degree of it vary depending on cultural standards of decency, honor, and face-saving (Edelmann, 1993; Markus and Kitayama, 1991). Authentic pride is valued in all cultures, but hubristic pride is more stigmatised, especially in collectivist societies where modesty is valued (Tracy et al., 2007; Kitayama et al., 2006). Pride is culturally nuanced. Although there are currently few systematic cross-cultural neuroimaging comparisons, neural studies also suggest that culture influences self-conscious emotional processing, especially in areas linked to social evaluation and self-construal (Zhu et al., 2007).

## 4 Discussion

Self-conscious emotions are significantly shaped by cultural variation. Comparative studies between Italian and American samples demonstrate the stark cultural differences between shame and guilt, with variations in their conceptual boundaries across societies (Giorgetta et al., 2023). On the other hand, pride is consistently acknowledged in a variety of cultural contexts, indicating that it is an expression that is evolutionarily universal (Tracy and Robins, 2008). This reliably recognized pride expression includes a slight upward head tilt, small smile, expanded chest and arms extended out from the body- either akimbo with hands on hips or raised above the head with hands on hips or raised above head with hands in fists (Mercadante et al., 2021). For embarrassment the nonverbal expression includes gaze down, followed by lip press, non-Duchenne smile and face touch, this was found reliably in American and Indian participants (Haidt and Keltner, 1999). As for shame head and gaze down with 54 and 64 AUs cross culturally recognized in US, England, Germany, Sweden, France, Greece, Japan and Burkino Faso (preliterate) (Izard, 1991; Keltner, 1995; Tracy and Robins, 2007). To date no research has found reliably recognizable nonverbal expressions of guilt.

Self-conscious emotions are also characterized by neural overlap and divergence, with numerous activating regions like the superior temporal sulcus (STS) and medial prefrontal cortex (MPFC). However, differences within these areas—dorsal MPFC in pride versus ventral MPFC in guilt, for example—highlight the distinct affective and cognitive processes that each emotion elicits (Takahashi et al., 2004; Gilead et al., 2016). Embarrassment has been mainly associated with the frontal and temporal lobes (Beer et al., 2003, 2006; Berthoz et al., 2002; Blair and Cipolotti, 2000; Devinsky et al., 1995; Ruby and Decety, 2004; Takahashi et al., 2004). Case studies have found that right-sided frontal lobe damage impairs understanding of embarrassing scenarios (Blair and Cipolotti, 2000). Guilt has been associated with diffuse activation in frontal and temporal lobes (Shin et al., 2000; Takahashi et al., 2004). Social Function of self-conscious emotions assert that these emotions evolved to serve specific adaptive functions (e.g., Ekman, 1992; Frijda, 1986; Johnson-Laird and Oatley, 1998).

Phenomenological studies of shame experiences also indicate that this emotion may be a signal of a threatened social self. Individuals have reported that they felt small, inferior to others, a sense of social isolation, and desire to hide from others in conjunction with shame experiences (Tangney et al., 1996). The elicitation of guilt is thought to arise in response to an undesirable behavior or action committed by an individual rather than to arise in response to an undesirable self, as in case of shame (Lewis, 1971; Tangney et al., 1996). Two forms of embarrassment have also been suggested, one occurring in response to nonevaluative social attention another occurring in situations where an actor is possible target of negative evaluation (Lewis and Ramsay, 2002). More empirical research is suggested to differentiate whether embarrassment can be characterized as part of a shame family of emotions or whether there are multiple forms of embarrassment. Pride is perhaps one of the least studied of all self-conscious emotions- a neglect that may be the result of a focus on negative emotions contributing to psychopathology and less historical interest in emotions that might promote positive mental well-being (Tracy et al., 2007, p. 72). There are several challenges in studying self-conscious emotions, which is why there is dearth of research in this area. This gap is caused by a number of conceptual and methodological issues. Firstly, Higher-order cognitive functions like moral reasoning, social comparison, and self-evaluation are all part of the intrinsic complexity of self-conscious emotions. They are challenging to induce in controlled laboratory settings because they frequently call for an internalized sense of self and awareness of social norms. Self-conscious emotions typically result from complex interpersonal contexts and subjective interpretations, in contrast to basic emotions, which are frequently elicited by simple stimuli (such as pictures of snakes or angry faces). Standardized, ecologically sound paradigms to consistently elicit self-conscious emotions in experimental settings are scarce. For instance, evoking true pride or shame frequently entails manipulating social judgment or personal failure, which presents ethical issues and might not elicit the same reactions in different people. In addition to requiring social interaction or interpersonal feedback, embarrassment and guilt can be difficult to replicate using scripted tasks or static stimuli. Secondly, it is challenging to measure self-conscious emotions objectively. They mainly rely on self-report, which may be skewed by individual differences in emotional granularity, introspective limitations, or social desirability bias. Compared to basic emotions, the physiological and neural markers linked to these emotions are also less clear. For instance, the neural substrates of shame or guilt are more dispersed and vary from study to study, whereas fear is consistently linked to amygdala activity.

In order to study self-conscious emotions, it is frequently necessary to manipulate participants’ moral standing or sense of self-worth, which can have long-lasting psychological effects. The need for experimental realism and ethical responsibility must be carefully balanced by researchers. This restricts the degree and genuineness of emotion that can be evoked in laboratory settings, frequently leading to mild or fabricated emotional experiences.

It is important to point out the conceptual blind spots in the field in addition to summarising the existing work. The majority of research ignores the overlaps and situational co-activation of self-conscious emotions, seeing them as distinct categories. Theoretical accuracy and experimental validity could be improved by creating integrative models that capture common self-evaluative mechanisms.

## 5 Limitations

Despite offering a thorough synthesis of empirical research on self-conscious emotions, this review has a few important limitations. First, the methodologies used in the reviewed studies differed greatly, with variations in measurement instruments, sample characteristics, and emotion induction strategies. This was the case also because of narrow inclusion and broad exclusion criteria since there is already very little empirical and experimental work in these emotions. Second, Western, Educated, Industrialized, Rich, and Democratic (WEIRD) populations are the subject of a disproportionate amount of the literature currently in publication. Therefore, it is important to exercise caution when making cross-cultural generalizations, particularly with regard to neural and physiological findings. Few studies used systematic cross-cultural designs or culturally sensitive methodologies, despite the fact that some examined cultural variability. Third, despite being instructive, the neuroimaging studies frequently had small sample sizes and used a variety of analytical techniques. As a result, certain neural correlates linked to each emotion are less reliable and reproducible. Furthermore, the present review only focused on the four canonical self-conscious emotions such as shame, guilt, embarrassment and pride. Although, Other self-referential emotions like humiliation (Hartling and Luchetta, 1999), feeling offended (Poggi and D’Errico, 2018), regret and remorse (Bhushan et al., 2020a, 2020b), and envy or jealousy (Smith and Kim, 2007) share underlying mechanisms linked to social appraisal, self-image and self-evaluation, they did not meet inclusion criteria. Future studies may benefit from taking these emotions into account as a component of larger self-conscious emotional system that includes relational, moral and reputational aspects.

## 6 Conclusion

Self-conscious emotions play crucial roles in regulating social behavior, moral decision-making, and self-evaluation. They emanate from one’s assessment of actions, traits, or behaviors in relation to societal norms and personal standards. Despite sharing core features such as self-referential processing and social salience, each emotion displays unique affective profiles, physiological responses, neural correlates, and non-verbal expressions.

Shame and guilt, though often conflated, differ meaningfully in their motivational direction and neural substrates. Embarrassment serves as a social appeasement signal, while pride, particularly authentic pride, functions to reinforce achievement and status. Cultural context further shapes how these emotions are experienced, expressed, and interpreted, underscoring the importance of culturally informed research.

Future studies should prioritize cross-cultural and longitudinal designs, integrate multimodal measures (e.g., physiological, neural, behavioral), and consider the dynamic interplay of multiple self-conscious emotions in complex social settings. Such approaches will deepen our understanding of how these emotions function across contexts and cultures, and how they contribute to both personal development and social cohesion.

### 6.1 Implication and future directions

The results of this review demonstrate the theoretical and practical significance of self-conscious emotions in influencing moral judgment, identity formation, and social behavior. These emotions, in spite of their complexity, are essential to comprehending how people behave in social and cultural settings. The unique contributions of shame, guilt, embarrassment, and pride to emotional experience and interpersonal dynamics are indicated by their different affective, physiological, and neural profiles. Nonetheless, a more methodical and comprehensive research agenda is required due to the present gaps in empirical evidence. The creation of ethically sound and ecologically sound techniques for eliciting self-conscious emotions in controlled environments should be the top priority of future research. While keeping experimental control, virtual reality, immersive storytelling, and interactive simulations may be useful tools for simulating authentic social contexts. These The ethical restrictions on evoking feelings like guilt or shame may also be circumvented with the aid of these technologies. The validity and reliability of results will be increased by combining self-report data with behavioral (such as posture, facial expressions), neurological (such as fMRI, EEG), and physiological (such as skin conductance, heart rate variability) indicators. Additionally, multimodal approaches can assist in separating the common and unique mechanisms that underlie basic emotions and self-conscious emotions.

To better understand how self-conscious emotions are experienced, valued, and expressed in various societies, more cross-cultural research is required. Generalisability is limited by the current literature’s strong bias toward Western samples. Clarifying how cultural norms, values, and language shape emotional processes will be made easier by extending research to non-Western, collectivist, and Indigenous cultural contexts.

## Data Availability

The original contributions presented in the study are included in the article/supplementary material, further inquiries can be directed to the corresponding author.
